# Investigating the correlation between tea intake and glioblastoma: A Mendelian randomization study

**DOI:** 10.1097/MD.0000000000042788

**Published:** 2025-06-13

**Authors:** Chaochao Zhang, Shuanchi Wang, Da Shi, Weidong Zhao

**Affiliations:** a Departments of Spine, Hebei Province Cangzhou Hospital of Integrated Traditional and Western Medicine, Cangzhou, China; b Hebei Key Laboratory of Integrated Traditional and Western Medicine in Osteoarthrosis Research, Cangzhou, China; c Departments of Neurology, Hebei Province Cangzhou Hospital of Integrated Traditional and Western Medicine, Cangzhou, China.

**Keywords:** causal association, glioblastoma, Mendelian randomization, single nucleotide polymorphisms, tea intake

## Abstract

Glioblastoma is a highly aggressive brain tumor with poor prognosis. Epidemiological studies suggest lifestyle factors, including tea intake, might influence glioblastoma risk. This study investigates the causal relationship between tea intake and glioblastoma risk using 2-sample Mendelian randomization (MR) analysis. Summary data from genome-wide association studies were used for MR analysis, employing single nucleotide polymorphisms (SNPs) associated with tea intake as instrumental variables. The causal effect of tea intake on glioblastoma risk was estimated using inverse-variance weighted (IVW), MR-Egger, weighted mode, and weighted median methods. Heterogeneity and pleiotropy were assessed using Cochran *Q* test and MR-Egger regression intercept. Forty SNPs were identified as instrumental variables. The IVW method showed no evidence of a causal association between tea intake and glioblastoma risk (OR = 0.6; 95% CI = 0.02–18.21; *P* = .7768). Supplementary analyses using MR-Egger, weighted median, and weighted mode methods were consistent with the IVW results. Leave-one-out analysis confirmed result stability, and the funnel plot indicated no pleiotropy. This MR analysis does not support a causal relationship between tea intake and glioblastoma risk. Rigorous methods are crucial for assessing causality in observational studies. Further research is needed to explore the impact of tea intake on glioblastoma using diverse study designs.

## 1. Introduction

Glioblastoma poses a significant problem in the field of neuro-oncology due to its unrelenting progression and limited effectiveness of treatment.^[1]^ The median survival for patients, despite aggressive multimodal therapy, remains dreadfully short.^[2]^ This depressing fact drives the pursuit of innovative prevention strategies that could potentially alter the course of this devastating disease. Amongst various lifestyle factors investigated for their roles in carcinogenesis, tea intake has attracted considerable interest because of its widespread prevalence and potential bioactive qualities that may have anticarcinogenic effects.

Its intake has been tentatively linked with reduced risks of several cancer types, posited to arise from the complex milieu of polyphenolic compounds with antioxidant and anti-inflammatory actions.^[3–6]^ Still, there is disagreement in the scientific community, particularly when it comes to high-grade gliomas like glioblastoma, where data is sparse and often contradictory.^[6,7]^ This uncertainty highlights the need for a methodologically robust approach to analyze the potential association between tea intake and glioblastoma incidence.

This study aims to leverage the power of Mendelian randomization (MR) to determine whether a causal relationship exists between tea intake and the risk of developing glioblastoma. By utilizing genetic variants as instruments for tea consumption, our analysis intends to bypass the confounding variables that often complicate observational studies. The hypothesis being tested is that increased tea intake, as predicted by genetic predispositions, is causally associated with a reduced risk of glioblastoma development.

To test this hypothesis, we analyze genetic data from large-scale genome-wide association studies (GWAS) that identify variants associated with tea intake using an MR framework.^[8]^ These genetic proxies serve as instrumental variables (IVs), offering a means to assess the relationship between tea consumption and glioblastoma risk in a manner grounded in causality rather than mere correlation.

Our study aims to provide clarity on the potential causal link between tea intake and glioblastoma risk, enhancing understanding of dietary influences on cancer prevention. A positive finding could reshape public health directives and invigorate preventative strategies against glioblastoma, potentially extending beyond the domain of neuroscience into the broader scope of cancer prevention. Furthermore, this study is set to showcase the applicability of MR in evaluating lifestyle choices as modifiable risk factors for cancer, paving the way for further research that could ultimately transform clinical practices and improve patient outcomes.

In sum, our MR study is poised to critically assess the causal effect of tea intake on glioblastoma risk. We anticipate contributing substantive evidence to the discourse on glioblastoma prevention, with the wider objective of informing both future research and public health strategies.

## 2. Materials and methods

### 2.1. Data source

In this study, a 2-sample MR approach was utilized to investigate the potential relationship between tea consumption and the risk of glioblastoma. The primary exposure variable was tea intake, while the outcome variable was glioblastoma incidence.

Genetic data related to tea intake were derived from GWAS within the UK Biobank, specifically using GWAS ID: ukb-b-6066. Tea intake was treated as a continuous variable, quantified as the number of cups consumed per day, with a mean value of 3.49631 cups/day and a standard deviation of 2.84255 cups/day. Data collection on tea consumption utilized 2 standardized touchscreen questionnaires that were validated by trained dietitians. The evaluation process was conducted centrally, ensuring consistency in data collection methods across participants: “Screenshot from the touchscreen questionnaire used to capture field 1488, Res ID 100318” and “Touchscreen questionnaire ordering, validation, and dependencies, Res ID 113241.” The questionnaires included the question: “How many cups of tea do you drink each day? (Include black and green tea),” with response options ranging from 0 to 99 cups. The mean tea intake reported was approximately 3.50 cups per day, with variations in individual consumption patterns. Participants were asked to report their average daily tea intake based on their consumption over the preceding year.

This dataset included a large cohort of 447,485 individuals of European ancestry and encompassed 9851,867 single nucleotide polymorphisms (SNPs). For missing data on self-reported tea intake, statistical imputation methods may have been applied to ensure data completeness and accuracy. This robust dataset provided a solid foundation for assessing the potential causal link between tea consumption and the risk of glioblastoma.

For the outcome data, we turned to the FinnGen biobank, which offered a GWAS dataset specific to glioblastoma under the GWAS ID: finn-b-C3_GBM. This dataset included a control group of 218,701 individuals, with a comprehensive set of 16,380,466 SNPs.

### 2.2. Study design

**Figure 1.**
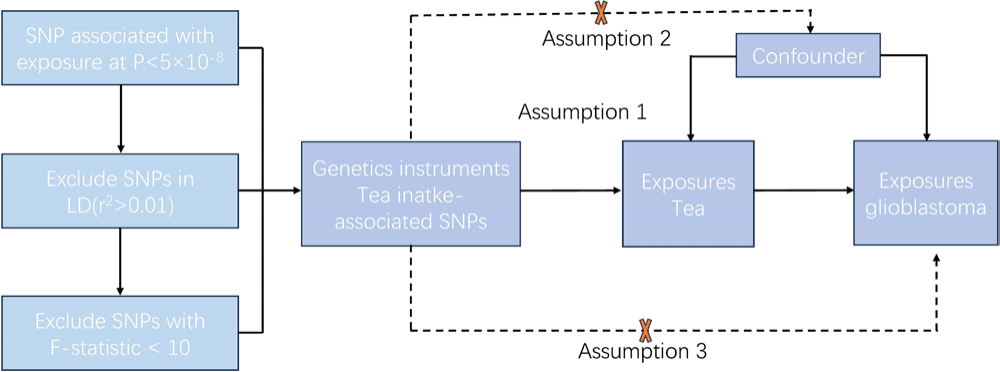
The design flowchart overview of MR study. MR = Mendelian randomization.

In this study, a bidirectional MR approach was employed to investigate the causal relationship between tea intake and the risk of glioma. The MR study is based on 3 core assumptions: the SNPs are strongly associated with the exposure (tea intake); there is no correlation between SNPs and confounding factors; the SNPs exert their effect on the outcome (glioma) through exposure (tea intake), that is, there is no gene pleiotropy.^[9,10]^ The design flowchart overview of this MR study is shown in Figure 1.

### 2.3. Data processing

Instrumental variables related to the exposure (tea intake) were identified using a threshold of *P* < 5 × 10^−8^. Subsequent filtering was carried out to eliminate SNPs within a 10 MB range that had *P*-values larger than .001, based on parameters of linkage disequilibrium with distance > 10,000 kb, *r*^2^ < 0.001, to remove SNPs in linkage disequilibrium and ensure the independence of the selected IVs. A secondary screening was then conducted to exclude variants associated with glioblastoma using a threshold of *P* < 5 × 10^−8^. Finally, the *F*-statistic (*F* = Beta^2^/SE^2^)was calculated, where an *F* > 10 indicates that the IVs are unlikely to be affected by weak instrument bias.^[11]^

### 2.4. Causal effect estimation

The primary method of analysis in this study was inverse-variance weighting (IVW), robust causal inference capabilities.^[12]^ However, the IVW method specifically requires genetic variation to only affect outcomes through exposure. Despite the rigorous exclusion of known confounding-related SNPs, the potential for bias due to unknown confounders remains. Consequently, MR-Egger, weighted median, and weighted mode were also applied as alternative MR analytical tools.^[13,14]^ A *P*-value of < .05 was considered to be statistically significant. If these 4 distinct MR methodologies converge on similar causal inferences, we consider the result to be robust and reliable.

### 2.5. Statistical analysis

We assessed the heterogeneity among SNPs using Cochran *Q* test and *I*^2^ statistics (*I*^2^ = 100%*(*Q*-df)/*Q*).^[15]^ If no significant heterogeneity was observed (*P* > .05), a fixed-effects model was utilized. Otherwise, a random-effects model was employed. To evaluate multiplicity, we employed multiple methods: we used MR-Egger intercept as an indicator of horizontal pleiotropy, the closer the number of the MR-Egger intercept to zero, the lower the probability of horizontal multiplicity. Sensitivity analysis was conducted using the “leave-one-out” approach to assess the influence of individual SNPs on the causal relationship.^[16,17]^

All statistical analyses were performed using the MR Base platform (App version: 1.4.3 8a77eb [October 2020], R version:4.0.3).^[16]^ *P*-value < .05 was considered statistically significant for differences.

## 3. Results

From the GWAS data of tea intake, 41 exposure related SNPs were obtained by screening with *P* < 5 × 10^−8^ as the standard and subsequently, SNP markers in linkage disequilibrium were excluded based on 2 parameters: kb > 10,000, *r*^2^ < 0.001. Further analysis was conducted on glioma cancer GWAS data, where SNPs associated with glioblastoma were excluded using a *P*-value threshold of 5 × 10^−8^, and 40 SNPs were finally screened. The *F*-statistic > 30 for each single variant, indicating that there is unlikely to be weak instrumental bias in the instrumental variable.

**Figure 2.**
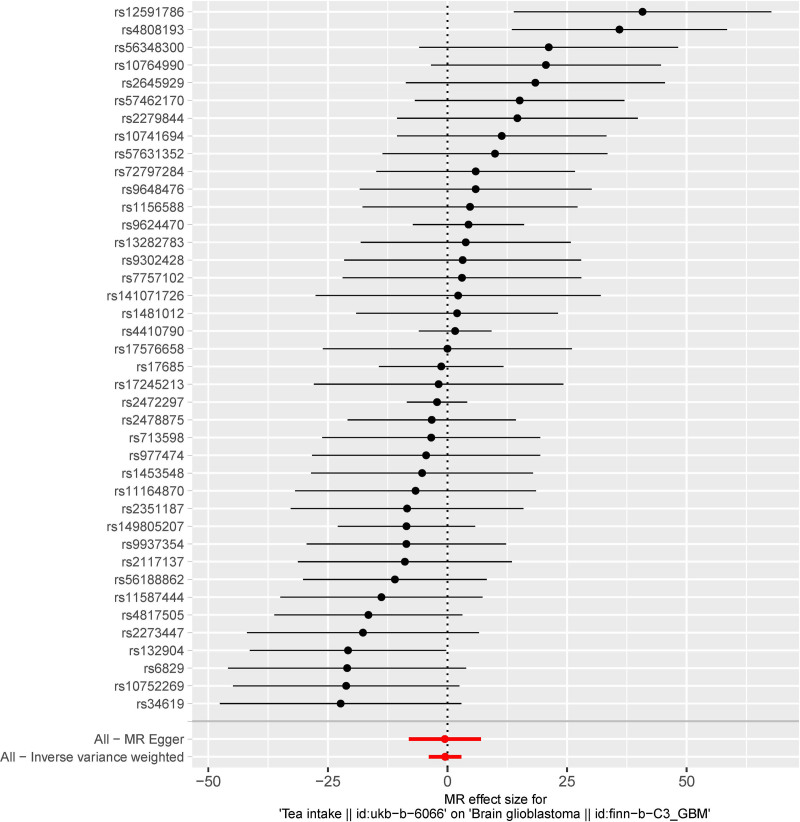
The forest plot of MR analysis for 40 single nucleotide polymorphisms. MR = Mendelian randomization.

**Figure 3.**
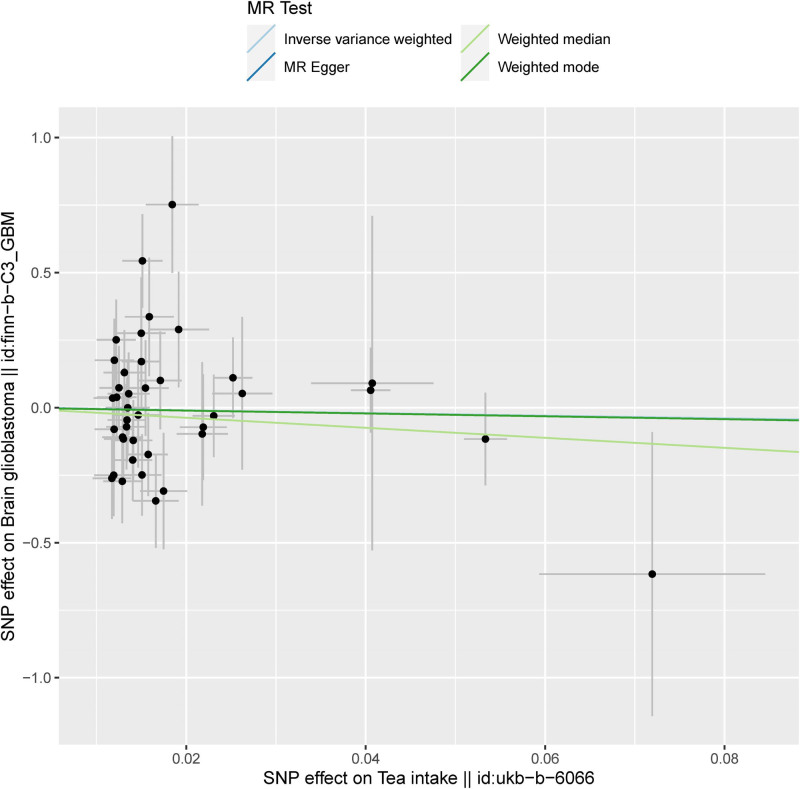
The scatter plot of 4 MR methods. MR = Mendelian randomization.

A causal relationship was observed between tea intake and glioblastoma (Table 1, Figs. 2 and 3). The IVW method showed no evidence to support a causal relationship between tea intake and glioblastoma (standard error [SE] = 1.731, *P* = .7768). MR-Egger analysis did not find any evidence of a causal relationship between tea intake and glioblastoma (SE = 3.846, *P* = .8881). The weighted median method also indicated no causal relationship between tea intake and glioblastoma (SE = 2.138, *P* = .3848). The weighted mode showed no causal relationship between tea intake and glioblastoma (SE = 2.328, *P* = .8212). The results of the above 4 analysis methods are consistent. Therefore, there is no causal relationship between tea intake and glioblastoma. The causal effects SNP associated with tea intake on glioblastoma are shown in Figure 2.

**Table 1:** Causal associations between tea intake and GBM.

MR method	Beta	SE	OR	95% confidence interval	*P*‐value
Inverse-variance weighted	−0.491	1.731	0.612	0.0206–18.210	.777
MR-Egger	−0.544	3.846	0.580	0.001–1089.594	.888
weighted median	−1.858	2.138	0.156	0.002–10.103	.383
Weighted mode	−0.530	2.380	0.589	0.006–56.446	.821

GBM = glioblastoma, MR = Mendelian randomization, OR = odds ratio, SE = standard error.

The sensitivity analysis showed that there were heterogeneities (IVW: *Q*-value = 56.86; df = 38; *P* = .0252; MR-Egger: *Q*-value = 56.86; df = 39; *P* = .03221); but no directional pleiotropies (MR-Egger intercept = 0.0011; SE = 0.073; *P* = .988).

**Figure 4.**
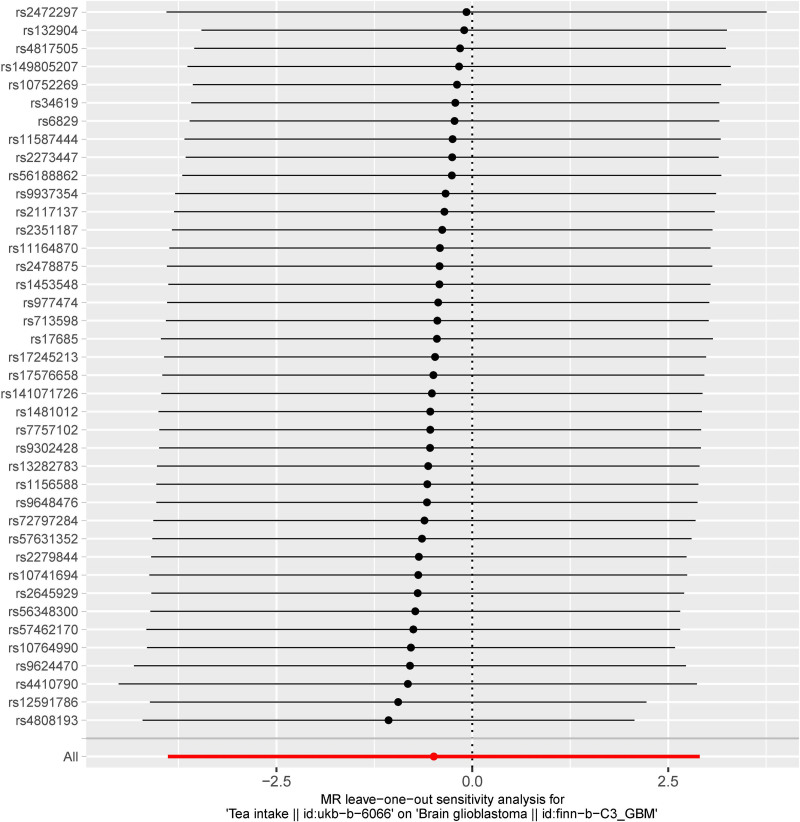
The funnel plot of MR analysis. MR = Mendelian randomization.

**Figure 5.**
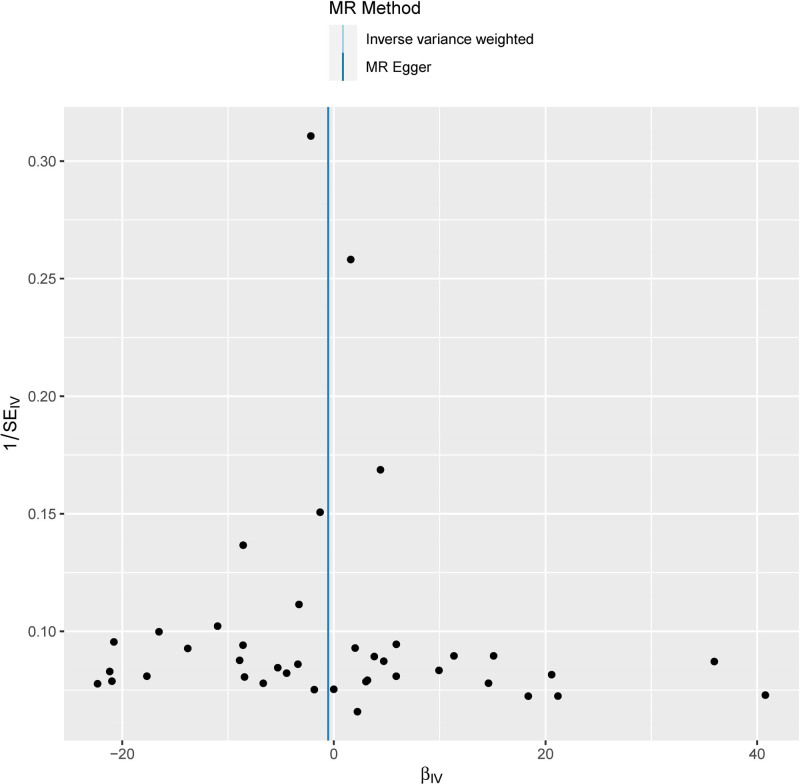
The forest plot of leave-one-out analysis.

Furthermore, the dataset filtered through MR-PRESSO did not exhibit any significant external perturbations, thereby bolstering the reliability of the results of this study. Constructing a leave-one-out plot revealed that removing any single IV did not result in significant deviation, maintaining consistent MR estimations. This indicates that the outcomes are not disproportionately influenced by any specific IV, further enhancing the robustness of the results (Fig. 4). The funnel plot demonstrated the symmetry of MR estimates, showing no signs of publication bias, which further supports the solidity and dependability of the research findings (Fig. 5).

## 4. Discussions

Understanding the risk factors for glioma holds paramount importance for devising effective prevention and treatment strategies. In our quest to examine lifestyle factors that may influence the risk of developing glioma, notably glioblastoma, tea intake emerged as a subject of interest due to its worldwide popularity and rich composition of bioactive compounds. Despite tea’s widespread acclaim for its health benefits, our MR analysis, leveraging genetic variants associated with tea intake, suggests that the relationship between tea intake and glioblastoma risk may not be causal.

Tea is a complex botanical concoction consumed globally, revered not only for its variety and taste but also for the multitude of bioactive compounds it harbors. These compounds, particularly catechins like epigallocatechin gallate (EGCG) found in green tea, have attracted significant attention for their potential antioxidative and anticancer properties, including liver cancer, biliary cancer, colorectal cancer, gastric cancer, and glioblastoma in vivo or in vitro.^[7,18–21]^ Scientific investigations into these constituents have highlighted several mechanisms through which they might mitigate cancer risk: Antioxidant Activity: EGCG is known to reduce oxidative stress by scavenging free radicals and enhancing the body’s antioxidant defense systems. This reduction in oxidative damage may lower the likelihood of mutations that could lead to cancerous transformations in cells.^[22]^ Inhibition of tumor proliferation: catechins have been shown to inhibit cell proliferation by interfering with signaling pathways critical for cancer cell growth, such as the PI3K/Akt and MAPK pathways.^[23]^ This interference can lead to reduced tumor growth and spread. Induction of apoptosis: green tea catechins can induce apoptosis, or programmed cell death, in cancer cells.^[24]^ They activate apoptotic pathways and inhibit antiapoptotic proteins, thereby promoting the destruction of malignant cells. Effects: chronic inflammation is a known risk factor for cancer development. EGCG exhibits anti-inflammatory properties by downregulating pro-inflammatory mediators and cytokines, potentially reducing the inflammatory environment that supports tumor growth.^[25]^ Inhibition of angiogenesis: the formation of new blood vessels, or angiogenesis, is crucial for tumor growth and metastasis. EGCG can inhibit angiogenesis by suppressing vascular endothelial growth factor and other angiogenic factors, thereby restricting the nutrient supply to tumors.^[26]^ Well-documented antioxidative and anti-inflammatory properties of green tea catechins, particularly EGCG, underscore the importance of further research into their potential roles in cancer prevention, though our findings do not support a direct causal relationship with glioblastoma.

Black tea, derived from the leaves of the Camellia sinensis plant, contains bioactive polyphenols such as theaflavins and thearubigins, which have been extensively studied for their various health benefits, particularly their potential anticancer properties. The mechanisms through which black tea exerts these effects include: Antioxidant activity: the polyphenols in black tea neutralize free radicals, thereby reducing oxidative stress and DNA damage associated with cancer initiation.^[27]^ Anti-inflammatory effects: by suppressing inflammatory pathways, black tea may lower the cancer risk linked to chronic inflammation.^[28]^ Inhibition of tumor growth: polyphenols disrupt critical signaling pathways, which slows cancer cell proliferation and induces apoptosis.^[29]^ Recent umbrella reviews have indicated that black tea consumption may be associated with a decreased risk of glioma, suggesting a potential protective effect against this aggressive form of brain cancer.^[30]^

In a systematic review and meta-analysis of tea consumption, it was found that individuals consuming 3 to 4 cups of tea per day exhibited a reduced risk of glioma (risk ratio = 0.84; 95% CI = 0.71–0.98), emphasizing the potential dose-response relationship; however, the duration of consumption in these studies was not consistently reported.^[6]^ Two previous studies, 1 conducted by the National Institutes of Health using the AARP cohort and the other involving Iranian adults of a case-control study have consistently demonstrated a significant inverse relationship between tea intake and the risk of developing glioma.^[31,32]^ However, 2 prospective studies from the UK Biobank and Japan respectively revealed a significant inverse relationship between tea intake and the risk of developing glioma.^[33]^ These findings stand in contrast to the results of our MR analysis, which did not corroborate a causal link between tea intake and a decreased risk of glioblastoma. This discrepancy underlines the complexities of dietary studies, where variables such as the types of tea, preparation methods, and individual genetics may influence outcomes.

Observational studies, while informative, are often limited by their inability to conclusively prove causality. The challenge lies not only in translating the antineoplastic effects observed in laboratory settings to human populations but also in determining the optimal quantities of tea intake for cancer prevention. Our investigation through the MR approach aimed to address these limitations by assessing the genetic predispositions that influence tea intake and their associations with glioma risk. Despite the lack of a significant causal linkage, our findings contribute to the ongoing discourse on dietary influences on cancer risk, underscoring the need for caution in interpreting observational data.

The absence of a causal relationship in our analysis could reflect the intricate interactions between the bioactive components of tea and the human body, further complicated by the multifactorial nature of glioblastoma pathogenesis. Additionally, the potential for pleiotropic effects not accounted for in our analysis, or the failure of our genetic instruments to capture tea intake variability across populations, cannot be overlooked. However, the robustness of our genetic instruments, as indicated by the MR-Egger intercept, lends confidence to our findings.

This study did not assess cumulative lifetime intake or differentiate effects of short-term versus long-term consumption. This study did not differentiate between black and green tea intake or account for preparation methods (e.g., brewing time), which may influence bioactive compound availability. While experimental data suggest bioactive components in tea may exert antineoplastic effects, our MR analysis specifically addressing causality found no evidence that genetically proxied tea intake alters glioblastoma risk. This opens avenues for future research to explore other modifiable risk factors and delve deeper into the biological underpinnings of glioma development. Further studies are warranted to dissect the complex interplay between dietary factors like tea intake, genetic predispositions, and glioma risk. Such research should aim to provide a more nuanced understanding of how different tea varieties, brewing methods, and consumption levels might interact with individual genetic backgrounds and environmental factors to influence cancer risk.

## 5. Limitations and future directions

Mendelian randomization studies have their limitations. The validity of IVs assumes that they are solely associated with the exposure (tea intake) and influence the outcome (glioblastoma) only through the exposure. However, the availability of suitable IVs is often limited, and the assumptions underlying MR should be carefully evaluated.

## 6. Conclusions

This MR study did not find evidence of a causal relationship between tea intake and glioblastoma risk. It underscores the importance of employing rigorous methods, such as MR, to assess causality in observational studies. While tea intake may offer other health benefits, its impact on glioblastoma development requires further investigation using various study designs and incorporating a range of factors.

## Acknowledgments

The study was based on the data provided by UK Biobank and FinnGen database. We thank the participants and investigators who provided valuable genetic summary statistics for this study.

## Author contributions

**Data curation:** Chaochao Zhang.

**Methodology:** Chaochao Zhang, Shuanchi Wang.

**Resources:** Shuanchi Wang.

**Validation:** Da Shi.

**Visualization:** Da Shi.

**Writing – original draft:** Chaochao Zhang.

**Writing – review & editing:** Weidong Zhao.
